# The effect of fiber intake on the association of pesticide exposure and hypertension: a population-level study

**DOI:** 10.3389/fpubh.2025.1556699

**Published:** 2025-03-21

**Authors:** Haili Lai, Xiaoqin Xin

**Affiliations:** ^1^Department of Gynecology and Obstetrics, Ganzhou Maternal and Child Health Hospital, Ganzhou, Jiangxi, China; ^2^Department of Clinical Laboratory, Ganzhou People’s Hospital, Ganzhou, Jiangxi, China

**Keywords:** hypertension, pesticide exposure, fiber intake, interaction, risk

## Abstract

The association between pesticide exposure and the risk of hypertension is inconsistent. Moreover, little is known about the effects of dietary fiber intake on the association between pesticide exposure and the risk of hypertension. This study aimed to assess whether fiber intake alters the relationship between pesticide exposure and hypertension. The study included 14,218 American adult patients. Multivariate logistic regression analysis was used to assess the relationship between pesticide exposure and the risk of hypertension. We also examined the relationship between pesticide metabolites in urine specimens and hypertension. Participants were stratified based on their mean fiber intake (low fiber intake: <17 gm and high fiber intake: ≥17 gm). An interaction test between dietary fiber intake, pesticide exposure, and risk of hypertension was conducted. Exposure to pesticides increased the risk of hypertension in the crude and full-adjusted models, and their odds ratio (ORs) [95% Confidence Interval (CI)] were 1.40 (1.26–1.56) and 1.19 (1.05–1.34), respectively. The analysis of pesticide metabolites indicated that dimethyldithiophosphate was statistically associated with hypertension (OR = 1.01, 95% CI = 1.01–1.02). The association between pesticide exposure and hypertension was opposite among participants in low and high fiber intake groups, OR = 1.34, 95% CI: 1.14–1.58 vs. OR = 0.98, 95% CI: 0.80–1.20, respectively, which implied that a high fiber intake may decrease the risk of hypertension (interaction likelihood ratio test: *p* = 0.031). We are the first to report the role of fiber intake in pesticide exposure and the risk of hypertension.

## Introduction

Hypertension is a public health problem affecting 160 million adults in the United States and > 1 billion adults globally (1–3). The specific pathogenic factors in >80% of hypertensive cases remain unknown, and environmental factors, including diet, are thought to be the multiple factors. More recently, the effect of exposure to environmental chemicals such as pesticides and lifestyle, including dietary patterns, on the development or progression of hypertension has received increasing attention (4–6).

Millions of kilograms of pesticides are produced yearly (7, 8). According to 2022 data from the Food and Agriculture Organization of the United Nations, global pesticide imports and exports have significantly increased from 1990 to 2020. In 2020, the United States was the leading country in pesticide usage (9). Moreover, some studies confirmed that 90% of American adults have considerable pesticide metabolite levels in their urine (10, 11). Pesticides are used for various purposes, including controlling pests and weeds, eliminating harmful fungi, and ensuring the successful production and harvest of crops. However, they pose significant health risks, with studies linking pesticide exposure to diseases such as cancer, immune and nervous system damage, as well as negative effects on asthma, reproduction, and birth defects (12). Similarly, pesticide exposure has been shown to negatively impact psychiatric disorders, diabetes, and erectile dysfunction (13–15). A study reported that exposure to DDT in mice causes sustained increases in systolic blood pressure, potentially through the activation of the renin-angiotensin system, as evidenced by the attenuation of hypertension following ACE inhibitor treatment (16). Another study found that PCB126 contributes to endothelial dysfunction in human hypertension by stimulating vasoconstrictors and inhibiting nitric oxide release (17). These findings suggest an association between pesticide exposure and hypertension. However, the relationship between pesticides and the risk of hypertension has been inconsistent in recent years. Javeres et al. (4) indicated that long-term pesticide exposure increases the risk of metabolic disturbances and hypertension. However, Warembourg et al. (18) stated that after multiple tests, early pesticide exposure was not significantly associated with diastolic blood pressure in children. In addition, a cross-sectional study conducted in a West African adult population showed that pesticide exposure was associated with lower blood pressure levels in women (19). Therefore, the relationship between pesticide use and hypertension requires further exploration.

Changing dietary patterns is thought to be beneficial in many chronic diseases (20). Increasing epidemiological evidence suggests that dietary fiber supplements protect against cardiovascular diseases and hypertension (21–23). However, whether dietary fibers affect the relationship between pesticide exposure and hypertension remains unknown. In this cross-sectional study, we evaluated whether there was a relationship between pesticide exposure at home and hypertension in the general population using data from the National Health and Nutrition Examination Survey (NHANES) 2013–2018. We also explored the effect of fiber intake on this relationship.

## Methods

### Study population

Data were extracted from the NHANES, a national survey that collects health and diet information from the United States population. Detailed information about the design and data collection procedures can be obtained from an open website.^1^ All study protocols were approved by the National Center for Health Statistics Ethics Review Committee (protocols 98–12, 2005–06, 2011–17, and 2018–01), and all participants provided written informed consent. We collected data on 29,400 individuals from three consecutive NHANES cycles (2013–2014, 2015–2016, and 2017–2018) and restricted the age range to 20–80 years (*n* = 17,057). Data on missing pesticide exposure, daily fiber consumption, hypertension, and other critical information were excluded (*n* = 2,569). The final sample size included 14,218 individuals.

### Definition of hypertension

Hypertension was determined with the following items: If participants responded to the question, “Have you ever been told by a doctor or other health professional that you have hypertension (also known as high blood pressure)?” or answered “yes” to the question, of whether they reported using anti-hypertensive drugs or they have high biological measurements (systolic blood pressure ≥ 140 mm Hg and/or diastolic blood pressure ≥ 90 mm Hg) (24). Each participant underwent three consecutive blood pressure readings. This study used the average of these readings to define hypertension.

### Pesticide exposure

Pesticide exposure at home was based on past literature definitions and considered in response to questions from a self-report questionnaire. “Have any chemical products been used to control fleas, cockroaches, ants, termites or other insects in (your/his/her) home in the last 7 days?” (25). In addition, we collected information on pesticide metabolites, and in the absence of other covariates, 3,505 participants for chlorophenol metabolites and 3,117 participants for acephate and ethylenethiourea were available for analysis.

### Dietary fiber intake

Dietary intake was calculated following the Dietary Interviewers’ Procedure Manuals found in NHANES (26). Briefly, dietary fiber intake was obtained by trained reviewers through a 24-h recall survey, commonly used in large-scale surveys. We divided dietary fiber intake into two groups (>17 gm vs. ≤17 gm) based on the mean value of our study’s total population.

### Covariates

Potential variables were also included based on the results of previous studies (25). Variables included race, age, sex, body mass index (BMI), waistline, marital status, PIR, educational level, alcohol intake, serum calcium level, smoking status, work activity, and dietary factors (magnesium, energy, protein, calcium, and vitamin D). Race was classified as others, non-Hispanic black, Mexican American, other Hispanic, or non-Hispanic white. BMI was calculated from examination data as weight in kilograms divided by height in meters squared and categorized as ≥30 and < 30 kg/m^2^. PIR is the ratio of family income to poverty. Education was classified into three grades (less than high school, high school, and college or above). Alcohol intake was categorized into three grades (non-drinkers, heavy drinkers, and low-to-moderate drinkers). Marital status was categorized into two types (married or living with a partner and living alone). Serum calcium concentration was measured using the ion-selective electrode method (UniCel R DxC 800 Synchron). Current smokers were defined as those who had smoked >100 cigarettes in their lifetime and smoked every day for several days during the interview. Former smokers were regarded as those who had smoked >100 cigarettes in their lifetime but did not smoke at the time of the interview. Non-smokers are those who have smoked <100 cigarettes in their lifetime. Work activities were categorized into three grades (light, moderate, and vigorous work activities). Dietary magnesium, energy, calcium, protein, and vitamin D values were collected, similar to fiber.

### Statistical analyses

Statistical analyses were performed using the statistical software package R (The R Foundation)^2^ and Free Statistics software version 1.3. Normally distributed continuous variables are expressed as the mean (standard error, SE), and non-normally distributed continuous variables are expressed as the median. Categorical variables were presented as numbers (survey percentage, %). Multivariate logistic regression models were used to calculate odds ratios (ORs) and 95% confidence intervals (CIs). To assess the effects of the covariates, they were gradually adjusted in all three models. We adjusted for none in Model 1; we adjusted for sociodemographic factors in Model 2, including age, sex, and race/ethnicity; additional covariates, including family poverty to income ratio, marital status, dietary factors (magnesium, energy, protein, fiber, calcium, and vitamin D), work activity, education degree, smoking, BMI, waist circumference, alcohol intake, and serum calcium were adjusted in Model 3. Subgroup analyses were performed based on age (< 40, 40–60, >60 years), sex (male, female), race, obesity (< 30 kg/m^2^, ≥ 30 kg/m^2^), education level, PIR, smoking status, and alcohol intake. Potential effect modifications (interactions) were assessed using a likelihood ratio test. Statistical significance was set at *p* < 0.05.

## Results

### Baseline characteristics of participants

A summary of the participants’ characteristics is presented in Table 1. Among the 14,218 participants, 51.3% were female, and 1,511 (10.62%) had a history of pesticide use at home. The mean fiber intake was 17 gm/day. Participants who used pesticides at home were more likely to be older, have a lower PIR, and have a lower dietary intake.

**Table 1:** The characteristics of the study grouped by pesticide exposure in home.

Variables	Total (*n* = 14,218)	Pesticide unexposed (*n* = 12,707)	Pesticide exposed (*n* = 1,511)	*p*-value
Gender, *n* (%)				0.544
Female	7,291 (51.3)	6,505 (51.2)	786 (52)	
Male	6,927 (48.7)	6,202 (48.8)	725 (48)	
Age, Mean ± SD (years)	50.0 ± 17.6	49.7 ± 17.6	52.4 ± 17.3	< 0.001
Race, *n* (%)				< 0.001
Mexican American	2095 (14.7)	1862 (14.7)	233 (15.4)	
Non-Hispanic Black	3,062 (21.5)	2,642 (20.8)	420 (27.8)	
Non-Hispanic White	5,481 (38.5)	4,966 (39.1)	515 (34.1)	
Other Hispanic	1,482 (10.4)	1,310 (10.3)	172 (11.4)	
Other Race	2,098 (14.8)	1,927 (15.2)	171 (11.3)	
PIR, Mean ± SD	2.5 ± 1.6	2.5 ± 1.6	2.2 ± 1.5	< 0.001
Education level, *n* (%)				0.005
Less than high school	2,922 (20.6)	2,564 (20.2)	358 (23.7)	
High school	3,274 (23.0)	2,930 (23.1)	344 (22.8)	
College or above	8,022 (56.4)	7,213 (56.8)	809 (53.5)	
Marital status, *n* (%)				< 0.001
Married or live with partner	8,455 (59.5)	7,659 (60.3)	796 (52.7)	
Live alone	5,763 (40.5)	5,048 (39.7)	715 (47.3)	
BMI, Mean ± SD ((kg/m2))	29.6 ± 7.2	29.6 ± 7.1	29.8 ± 7.6	0.216
Waistline, Mean ± SD	100.6 ± 17.0	100.5 ± 16.8	101.3 ± 17.8	0.089
Serum calcium, Mean ± SD (mg)	2.3 ± 0.1	2.3 ± 0.1	2.3 ± 0.1	0.945
Magnesium (mg), Median (IQR)	268.0 (194.0, 368.0)	268.0 (194.0, 367.0)	269.0 (196.0, 369.5)	0.79
Energy (kcal), Median (IQR)	1939.0 (1423.0, 2588.0)	1932.0 (1420.0, 2574.0)	1997.0 (1470.0, 2706.0)	0.002
Protein (gm), Median (IQR)	72.8 (51.7, 100.8)	72.8 (51.7, 100.8)	72.7 (51.6, 99.8)	0.803
Fiber, Mean ± SD	17.0 ± 10.9	17.0 ± 11.0	16.4 ± 10.4	0.027
Calcium (mg), Median (IQR)	799.0 (513.0, 1169.0)	797.0 (513.0, 1166.0)	807.0 (510.5, 1194.0)	0.915
Vitamin D (mg), Median (IQR)	2.9 (1.1, 5.7)	2.9 (1.1, 5.7)	3.0 (1.2, 6.0)	0.252
Smoking status, *n* (%)				< 0.001
Never smoker	8,082 (56.8)	7,313 (57.6)	769 (50.9)	
Former smoker	3,423 (24.1)	3,027 (23.8)	396 (26.2)	
Current smoker	2,713 (19.1)	2,367 (18.6)	346 (22.9)	
Work activity, *n* (%)				0.005
Light	8,071 (56.8)	7,261 (57.1)	810 (53.6)	
Moderate	3,036 (21.4)	2,713 (21.4)	323 (21.4)	
Vigorous	3,111 (21.9)	2,733 (21.5)	378 (25)	
Alcohol intake, *n* (%)				0.516
Nondrinker	11,055 (77.8)	9,894 (77.9)	1,161 (76.8)	
Low-to-moderate drinker	973 (6.8)	871 (6.9)	102 (6.8)	
Heavy drinker	2,190 (15.4)	1942 (15.3)	248 (16.4)	
Hypertension, *n* (%)				< 0.001
No	8,641 (60.8)	7,833 (61.6)	808 (53.5)	
Yes	5,577 (39.2)	4,874 (38.4)	703 (46.5)	

### Association between pesticide exposure and the risk of hypertension

Table 2 shows the relationship between pesticide exposure and hypertension using univariate and multivariate logistic regression analyses. The non-insecticide-exposure group served as a reference. In the Crude Model and Model 1, participants exposed to household pesticides had a higher risk of hypertension than participants not exposed to household pesticides, and their ORs (95% CI) were 1.40 (1.26–1.56) and 1.22 (1.09–1.38), respectively. Further, in Model 3 (full adjustment model), the results remained statistically significant (OR = 1.19, 95% CI: 1.05–1.34, *p* = 0.008). We further explored the relationship between pesticide metabolites in urine specimens from the study population and the risk of hypertension. We found that dimethyldithiophosphate was statistically associated with a higher risk of hypertension (Table 3).

**Table 2:** Associations between household pesticide exposure and hypertension.

Pesticide exposure	Event (%)	Crude model		Model 1		Model 2	
		OR (95% CI)	*p*-value	OR (95% CI)	*p*-value	OR (95% CI)	*p*-value
Pesticide unexposed	4,874 (38.4)	1 (Ref)		1 (Ref)		1 (Ref)	
Pesticide exposed	703 (46.5)	1.40 (1.26–1.56)	<0.001	1.22 (1.09–1.38)	0.001	1.19 (1.05–1.34)	0.008

Ref, reference. Crude model adjusted for none. Model 1 adjusted for sex, age and race. Model 2 adjusted for gender, age, race, education level, marital status, BMI, PIR, waistline, alcohol intake, serum calcium, energy, protein, Vitamin D, calcium, fiber, magnesium intake, smoke status, and work activity.

**Table 3:** The associations between pesticide metabolites and hypertension.

Pesticide metabolites	OR	95% CI	*p-*value
Bisphenola	1	1.00–1.01	0.266
Bisphenolf	1	1.00–1.01	0.142
Bisphenols	1	0.98–1.01	0.722
Triclosan	1	1.00–1.00	0.103
Propyl paraben	1	1.00–1.00	0.09
Dimethyldithiophosphate	1.01	1.01–1.02	0.03
Meoh phthalate	1	1.00–1.00	0.22
Mono benzyl phthalate	1	1.00–1.00	0.27
Butyl paraben	1	1.00–1.00	0.94
X2.5 dichlorophenol	1	1.00–1.00	0.64
X2.4 dichlorophenol	1	1.00–1.00	0.53

OR, odds ratio; CI, confidence intervals. Adjusted for gender, age, race, education level, marital status, BMI, PIR, waistline, alcohol intake, serum calcium, energy, protein, Vitamin D, calcium, fiber, magnesium intake, smoke status, and work activity.

### Fiber intake alleviates the relationship between pesticide exposure and hypertension

Table 4 shows that, in three models, the association between pesticides exposure and hypertension risk consistently existed among participants with low fiber intake compared with the reference group (non-pesticide exposure) (Model 1:OR = 1.50, 95% CI: 1.31–1.72, *p* < 0.001; model 2: OR = 1.36, 95% CI: 1.17–1.59, p < 0.001; model 3: OR = 1.32, 95% CI: 1.11–1.55, *p* = 0.001). However, the association disappeared in adjusted models among these high fiber intake participants (*p* > 0.05). Furthermore, we observed an interaction of fiber intake with the association between pesticide exposure and hypertension risk in Model 3 (interaction likelihood ratio test: *p* = 0.031).

**Table 4:** Interactive effect of dietary fiber intake and pesticide exposure in home on hypertension.

Models	Low fiber intake (< 17 gm) *n* = 8,527		High fiber intake (≥ 17 gm) *n* = 5,691		*P* for interaction
	OR (95% CI)	*P*-value	OR (95% CI)	*P*-value	
Model 1					0.096
Pesticide unexposed	1 (Ref)		1 (Ref)		
Pesticide exposed	1.50 (1.31–1.72)	<0.001	1.24 (1.04–1.48)	0.014	
Model 2					0.037
Pesticide unexposed	1 (Ref)		1 (Ref)		
Pesticide exposed	1.36 (1.17–1.59)	<0.001	1.03 (0.85–1.25)	0.769	
Model 3					0.031
Pesticide unexposed	Reference		Reference		
Pesticide exposed	1.34 (1.14–1.58)	<0.001	0.98 (0.80–1.20)	0.852	

Ref, reference. Model 1: adjusted for none; Model 2: adjusted for age, sex and race/ethnicity; Model 3: adjusted for gender, age, race, education level, marital status, BMI, PIR, waistline, alcohol intake, serum calcium, energy, protein, Vitamin D, calcium, fiber, magnesium intake, smoke status, and work activity.

### Subgroups analysis

**Figure 1:**
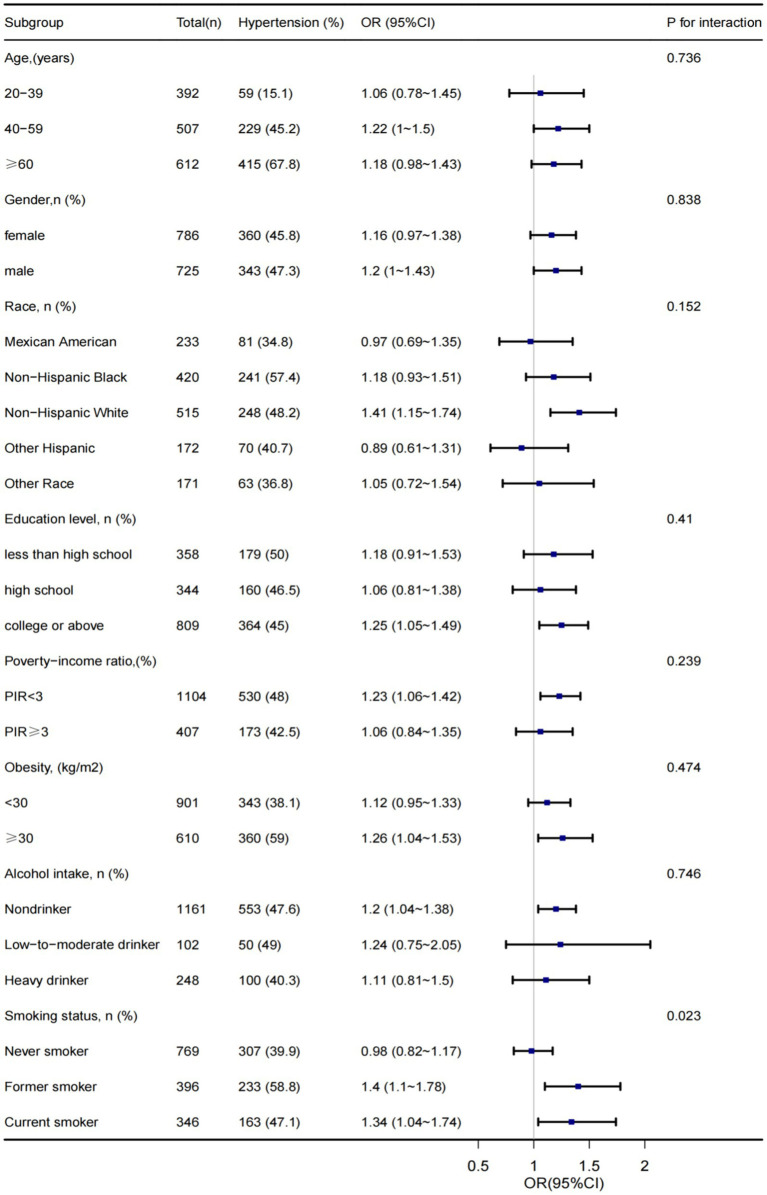
Subgroups analysis of association between household pesticide exposure and hypertension.

Subgroup analyses were stratified based on age, sex, race, obesity, education level, PIR, smoking status, and alcohol intake to ensure the stability of the results. After adjusting for potential variables, we found that, except for smoking status, other subgroups had non-interactive effects, which implied the harmful effects of smoking whenever possible (Figure 1).

## Discussion

We found that pesticide exposure at home was associated with the risk of hypertension in the general population. Furthermore, to our knowledge, this is the first report on the interaction between fiber intake and the association between pesticide exposure at home and the risk of hypertension. Our results suggest that dietary fiber intake may attenuate the harmful effects of pesticide exposure at home on the risk of developing hypertension.

Our results are consistent with those of a previous study that reported an association between organophosphate insecticide exposure and hypertension. A study indicated pesticide exposure was positively associated with the risk of hypertension (OR = 1.10, 95% CI: 1.01–1.18) (24). A similar observational study showed that pesticide exposure was an independent predictor of aortic diastolic and systolic blood pressures in 198 males (27). In addition, a cross-sectional study concluded that longer periods of spraying agricultural pesticides were associated with higher blood pressure among children living in agricultural communities in Ecuador (28). Similarly, data from two observational studies showed that potential pesticide exposure in the first trimester of pregnancy, including living in an agricultural area or engaging in agricultural activities, may increase the risk of high blood pressure in pregnancy (29, 30). Recently, a cohort study from Pakistan and Cameroon showed that elevated body mass index (BMI), insulin, blood sugar, dyslipidemia, and hypertension were observed in an organophosphorus (OPs) insecticide exposure group (4). In addition, data from systematic reviews and cohort studies have revealed that pesticides and pesticide metabolic exposure are linked to a higher risk of hypertension (31–34). Evidence from a systematic review and meta-analysis of 23 prospective studies suggested that exposure to the pesticide DDT possibly increases the risk of hypertension (35). However, most previous studies had a smaller sample size than ours, and our conclusions could extend to the general population. Animal studies have also reported similar results. Bataillard et al. (36) showed that the intravenous injection of organophosphorus, including paroxyphos and somans, led to a significant, sustained, and dose-dependent elevation of blood pressure in rats. Similarly, another rat model experiment suggested that exposure to chlorpyrifos might cause a sustained elevation in blood pressure (37).

Animal experiments and epidemiological studies have supported the link between pesticide exposure and hypertension; however, the exact underlying mechanisms remain unclear. Evidence suggests that oxidative stress leads to increased production of reactive oxygen species (ROS) and high concentrations of intracellular ROS, which impair vascular function and integrity and are vital in the pathogenesis of hypertension (38). Organophosphates induce oxidative stress, leading to abnormal blood pressure control (39). Recent studies have also shown that the pathogenesis of hypertension involves a gut microbiome imbalance (39). Pesticides have been shown to interfere with intestinal microenvironmental imbalances by upregulating cytokines (40, 41). Data from an adult rat model showed that exposure to dichlorodiphenyltrichloroethane (DDT) during pregnancy induces hyperactivation of the renin-angiotensin system, leading to hypertension and cardiac hypertrophy (16). An *in vitro* experiment demonstrated that exposure to chemicals such as polychlorinated biphenyls (PCBs) directly causes endothelial cell dysfunction (42). PCBs also increase cellular oxidative stress, subsequently increasing the risk of high blood pressure and cardiovascular diseases (43).

Previous studies have demonstrated that dietary fiber intake positively affects stroke prevention and hypertension management (44, 45). Increasing dietary fiber intake can significantly improve blood pressure in adults with hypertension, which can be explained by the role of dietary fiber in lowering LDL cholesterol and triglyceride uptake (46), which in turn increases vascular wall elasticity, reduces vascular resistance, and maintains adequate blood perfusion. Moreover, reports show that high fiber intake may increase antioxidants and reduce the role of oxidative stress in the pathogenesis of hypertension, Sinha and Dabla (47) and Baradaran et al. (48), which possibly explains the importance of high fiber consumption in restoring oxidative damage caused by pesticides. Our results showed that participants on a high-fiber diet had a lower risk of developing hypertension when exposed to pesticides at home. However, previous studies have ignored this crucial dietary factor, which may explain these inconsistent results.

The present study had several strengths. We included many men and women, a national representative of the U.S. adult population ranging from 20 to 80 years. In addition, we are the first to report an interaction between fiber intake and the association between pesticide exposure at home and risk of hypertension. Previous studies have targeted populations with occupational pesticide exposure, including pesticide applicators and individuals living in agricultural areas with high concentrations of pesticides. The study population was more representative, and the use of pesticides at home was explained. Subgroup analyses stratified by age, sex, race, PIR, BMI, and smoking status were performed to examine specific individuals.

This study has some limitations. First, owing to the nature of the cross-sectional study, a causal relationship between pesticide exposure and hypertension could not be clarified. Second, we used self-reported results to determine pesticide exposure outcomes; therefore, misclassification could have resulted from recall. Third, data on the type of pesticide, duration, and exposure concentration could not be obtained from NHANES. However, the association between pesticide metabolites in urine and the risk of hypertension was subsequently assessed. Our results showed that dimethyldithiophosphate was significantly associated with an elevated risk of hypertension.

## Conclusion

We found that pesticide exposure at home was correlated with the risk of hypertension in the general population. Furthermore, we are the first to report an interaction between fiber intake and the relationship between pesticide exposure and hypertension. Our results need to be further certified in future studies.

## Data Availability

The original contributions presented in the study are included in the article/supplementary material, further inquiries can be directed to the corresponding authors.

## Footnotes

^1^ https://www.cdc.gov/nchs/nhanes/index.htm

^2^ http://www.R-project.org
